# MR imaging findings of metastatic hepatocellular carcinoma in the nasal cavity: a rare site of spread

**DOI:** 10.1259/bjrcr.20160111

**Published:** 2016-11-03

**Authors:** Dong Hyeok Kang, Sang Woo Shim, Su Jin Koh, Jung Gwon Nam, Young Min Kim, Young Cheol Weon

**Affiliations:** ^1^Department of Radiology, Ulsan University Hospital, University of Ulsan College of Medicine, Ulsan, Republic of Korea; ^2^Department of Internal Medicine, Ulsan University Hospital, University of Ulsan College of Medicine, Ulsan, Republic of Korea; ^3^Department of Otorhinolaryngology, Ulsan University Hospital, University of Ulsan College of Medicine, Ulsan, Republic of Korea; ^4^Department of Pathology, Ulsan University Hospital, University of Ulsan College of Medicine, Ulsan, Republic of Korea

## Abstract

We here report an extremely rare case of metastatic hepatocellular carcinoma to the nasal cavity only with MRI scan including diffusion-weighted imaging and a brief review of previous literature case reports.

Metastatic tumours in the sinonasal region are relatively rare, and the most frequent primary site is the kidney, followed by the lung, breast, urogenital tract, gastrointestinal tract and thyroid gland.^1,2^ Extrahepatic metastasis of hepatocellular carcinoma (HCC) occurs in about 30–50% of patients and the most common sites are lung, lymph nodes, bone and adrenal gland.^3–5^ The sinonasal region, however, is an unusual site for metastatic HCC, and the most affected regions in order of decreasing frequency are the maxillary sinus, ethmoid bones and sphenoid bones.^6^ Metastases to the nasal cavity, including the nasal septum and turbinates, are even more rare; only 13 cases are reported in the literature (Table 1). These include eight patients with metastatic HCC involving the paranasal sinus and nasal cavity at presentation, four patients with involvement of the nasal septum or nasal vestibule, one patient with involvement of the nasal septum and nasal cavity, and three patients with involvement of only the nasal cavity. Herein, we present an additional case of rapidly growing metastatic HCC to the nasal cavity alone, with MRI scan and review of the reported cases.

**Table 1.** Thirteen reported cases of biopsy-proven metastatic HCC to the sinonasal region

No	Sex/age (y)	Sx	Carrier, risk factor	HCC dx to nasal sx	Extrahepatic mets	Involved site	Lung → SN mets	CT scan	MRI scan	Tx	Progrosis (after nasal mets)
Frigy	M/61	Epistaxis	Alcoholism	At presentation	Lung	Ethmoid sinus, nasal cavity	At presentation	No	No		Died suddenly, respiratory failure
Patankar	M/50	Epistaxis, nasal obstruction	NA	NA	Not mentioned	Nasal cavity		Mass	No	Refused tx	NA
English III	M/44	Epistaxis, facial pain	Hep C, S/P LT	NA	Not mentioned	Nasal cavity		Mass	No	Chemo	Bone metas, new hepatic HCC (several m)
Lin	M/45	Septal mass	Hep B	2 y 3 m	Duodenum	septum		No	No	RTx	Died, hepatic failure (6 w)
Matsuda	M/71	Epistaxis		7 y	Lung	Maxillary sinus, nasal cavity	6 m	Osteolysis of maxillary and orbit	No	RTx	Died, hepatic failure (8 w)
Chang	M/49	Septal mass	Hep B	16 m	Lung	Septum	16 m	Enhancing tumour	No	RTx	Aliver at 15 m follow-up
Kurisu	M/76	Nasal obstruction	Hep C	2 y	Bone	Maxillary sinus, nasal cavity		Mass	No	RTx	NA
Kurisu	M/69	Nasal obstruction	NA	NA	NA	Maxillary & ethmoid sinuses, nasal cavity		Mass	No		NA
Liu	M/55	Epistaxis	Hep B	1 y	Lung, mediantinum, LN	Vestibule, vault	1 w	Nasal cavity, maxillary bone erosion	No	Excisional biopsy	Brain mets, died, multiple metastatic disease (2 m)
Hwang	M/49	Epistaxis, nasal obstruction	NA	13 m	Heel	Septum, nasal cavity		Mass	Mass	Surgical resection	Aliver at 8 m f/u
Choi	M/45	Nasal mass		3 y	Lung, abd LN	Vestibule	1 y	Mass	No	Chemo	Aliver at 6 m f/u
Izquierdo	M/59	Epistaxis, nasal obstruction	Hep B, hep C	4 y	Not mentioned	Maxillary sinus, nasal cavity		CT	No		Died, hepatic failure during the hospitalization
Present case	M/53	Headache, nasal obstruction	Hep B	3 m	Lung	Nasal cavity	3 m	Osteolysis	Mass	Excisional biopsy	Discharged, awaiting next chemoTx

Abd, abdominal; Dx, diagnosis; f/u, follow-up; HCC, hepatocellular carcinoma; Hep, hepatitis; LN, lymph node; LT, liver transplantation; mets, metastasis; NA, not available; RTx, radiation therapy; Sx, symptoms; w, week; M, male; m, month; Tx, therapy; y, year

## Case report

**Figure 1.**
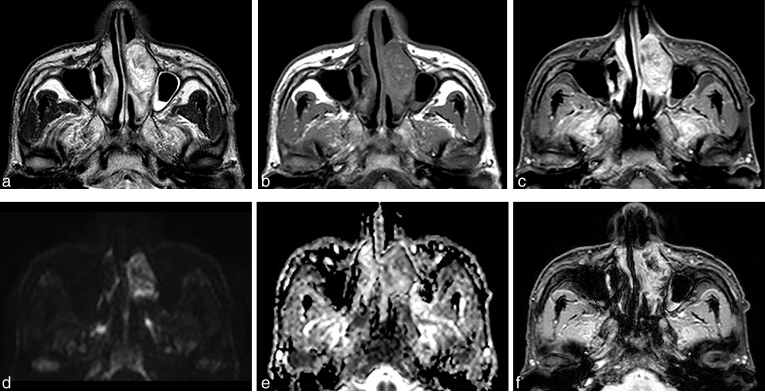
(a–f) MRI scan shows a mass occupying the left nasal cavity. (a) Axial *T*_2_ weighted MRI scan shows heterogeneous high- signal-intensity mass compared with muscle. (b) Axial *T*_1_ weighted MRI scan shows heterogeneous iso-signal-intensity mass and multifocal high-signal foci that suggest a haemorrhagical component. (c). Axial gadolinium-enhanced *T*_1_ weighted MRI scan shows heterogeneous enhancement except for multifocal haemorrhagical foci. (d, e) Axial DWI and ADC MRI scan shows that the mass has no diffusion restriction (average ADC value = 1536.01 mm^2^ s^−1^). (f). Axial gradient-recalled echo MRI scan shows heterogeneous high-signal-intensity mass and multifocal haemorrhagic foci with dark signal intensity (arrow). ADC, apparent diffusion coefficient; DWI, diffusion-weighted imaging.

**Figure 2.**
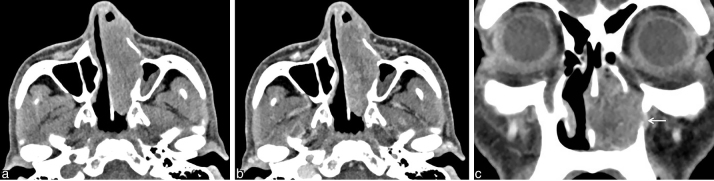
a–c. CT scan shows a mass occupying the left nasal cavity. (a) Axial noncontrast CT scan shows the iso-attenuating mass compared with muscle and no calcification. (b) Axial contrast-enhanced CT scan shows the mass is heterogeneously enhancing. (c) Coronal contrast-enhanced CT scan shows the mass occupying the left nasal cavity. The lateral wall of the maxillary sinus is osteolysed.

**Figure 3.**
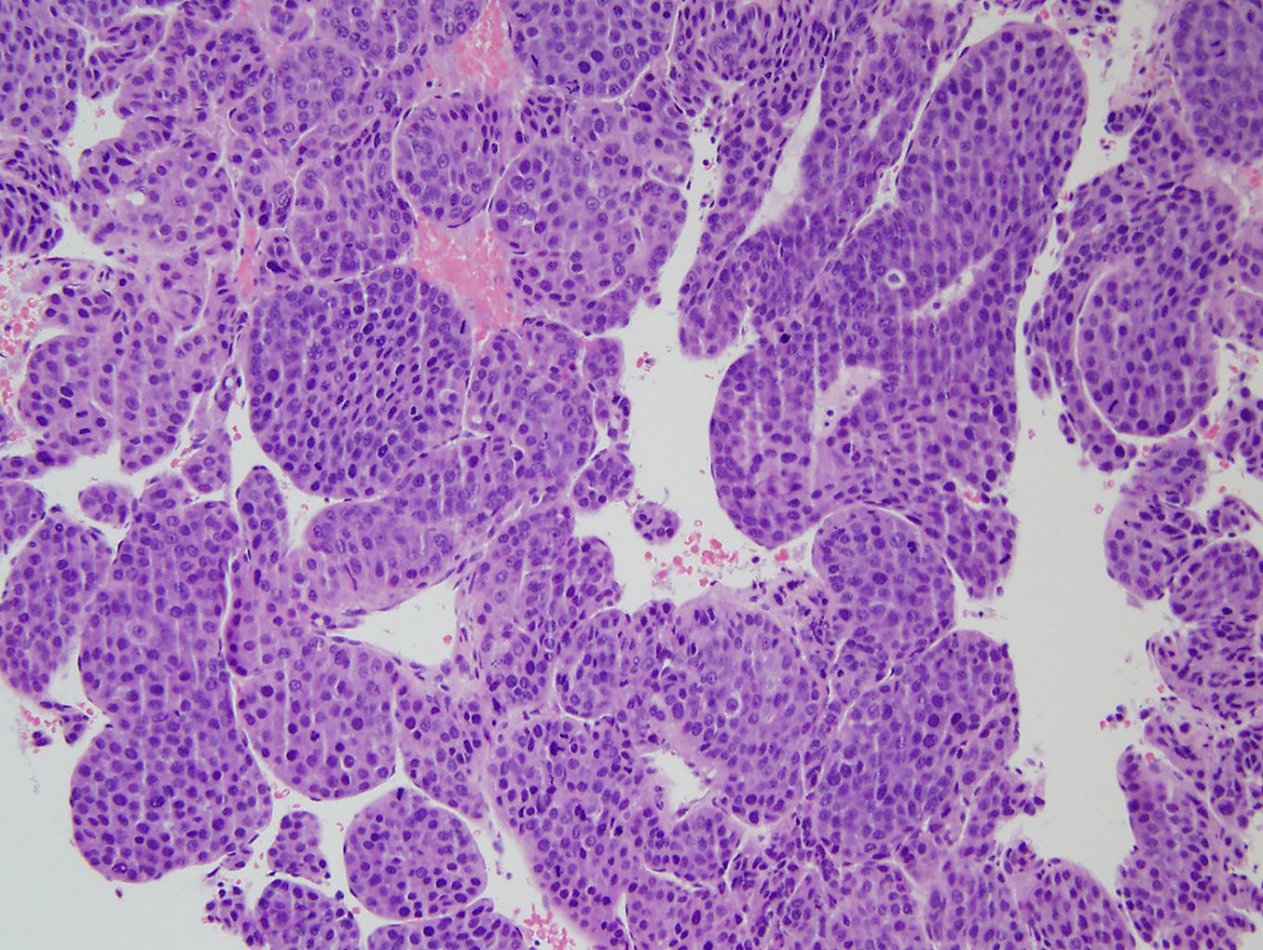
Pathological finding of the biopsy specimen from the left nasal cavity mass. Neoplastic polygonal cells are arranged in thick trabecular patterns, consistent with metastasis of hepatocellular carcinoma (hematoxylin and eosin stain; 200× magnification).

A 53-year-old male patient (hepatitis B carrier), who had been suffering for 3 months from HCC with multiple lung metastases, was admitted complaining of headache. A brain MRI scan that was performed to identify brain metastasis showed a solid mass occupying the left nasal cavity (3.7 × 1.8 × 2.8 cm). The mass showed iso-signal intensity on *T*_1_ weighted image, high signal intensity on *T*_2_ weighted image, and heterogeneous well enhancement on Gd-T_1_ weighted image (Figure 1). Gradient-echo images showed small foci of low signal, suggesting haemorrhage in the mass. Restricted diffusion was not noted in the mass on diffusion-weighted imaging (DWI) and apparent diffusion coefficient (ADC) map. Metastasis of HCC was suggested, as the nasal cavity was normal on a positron emission tomography CT scan that had been performed 3 months ago. The patient had developed left nasal obstruction, clear rhinorrhea and left facial pain. A CT scan that was performed for excisional biopsy (17 days after the MRI scan) showed that the mass had rapidly increased in size (6.2 × 2.2 × 3.4 cm) with involvement of the ostium of the nasolacrimal duct and the lateral wall of the anterior nasal cavity (Figure 2). There was no calcification in the mass on the pre-contrast CT scan. A punch biopsy was performed. Histological examination revealed tumour cells with enlarged nucleoli and clear cytoplasm arranged in trabecular cords and glandular arrays, consistent with metastatic HCC (Figure 3). The tumour was grade 2 (moderately differentiated). While the patient was awaiting resection of the tumour, his general condition declined and he was transferred to another hospital owing to his location.

## Discussion

HCC is the most common primary tumour of the liver, and its treatment depends entirely on the tumour stage and hepatocellular reserves.^3^ Knowledge of the location and radiographical appearance of metastatic HCC is therefore important for accurate tumour staging, to assure the patient the most appropriate treatment and best chance for survival. Because of rarity and lack of distinguishing features, metastasis of malignant neoplasms to the sinonasal region is often mistaken for primary neoplasm of the sinonasal tract.^7^ The kidney is the most common primary site, but other sources include the lung, breast, urogenital tract, gastrointestinal tract and thyroid gland.^1,2^ HCC easily metastasizes, and extrahepatic metastasis occurs in more than 50% of HCC patients, with the most common metastatic sites being the lungs, abdominal lymph nodes, bone and adrenal glands.^3–5^ Metastasis of HCC to the sinonasal region, however, is uncommon and metastasis to the nasal cavity is exceedingly rare, with only 13 cases reported in the literature (Table 1). Among them, eight patients had metastatic HCC involving the paranasal sinus and extending into the nasal cavity (the maxillary sinus in three cases, the ethmoid sinus in one case, and the maxillary and ethmoid sinuses in one case); five patients had involvement of the nasal septum or nasal vestibule; and three patients had involvement of only the nasal cavity. As CT scan is the primary choice for imaging study of the head and neck region, MRI scan of these lesions was performed in only one case.^8^ We present an additional case of rapidly growing metastatic HCC to the nasal cavity with MRI scan.

HCC metastasizes by either lymphogenous or haematogenous spread.^9^ It is frequently noted to invade the local vascular network by direct extension into the caval venous system.^10^ Haematogenous spread through the systemic circulation is thus readily explainable. Once the tumour emboli enter the vascular system, they can flow through the pulmonary circulation and reach the sinonasal area through the arterial system of the head and neck.^1,2,11,12^ Including our case, more than half of reported cases of metastatic HCC to the nasal cavity had lung metastasis at presentation. If there is no evidence of lung metastasis, it has been postulated that the disease can spread through Batson’s paraspinal venous plexus is a valveless venous system in the prevertebral, vertebral and epidural space.^2^ Without valves, the venous plexuses do not resist the spread of tumour emboli (especially during increase of intra-abdominal or intrathoracic pressure)^13^ and allow metatastic emboli to bypass to the pulmonary venous system, giving rise to metastasis to the head and neck region without involvement of the lungs.^2,13^ The lymphatic system provides another route of spread. Tumour emboli from the regional lymph nodes can flow into the thoracic duct. In such cases, invasion of the hepatic, peripancreatic, celiac and para-aortic lymph nodes would be expected before the disease would spread into the head and neck.^14^ Metastases can reach the head and neck via retrograde flow through the intercostal, mediastinal or supraclavicular lymph vessels.^15^ In our case, because there was no metastatic lymphadenopathy but lung metastases were present, lymphatic spread seems more likely than haematogenous spread.

Metastatic tumours to the sinonasal cavity have no distinctive clinical features that may facilitate their early diagnosis. Epistaxis, facial deformity, pain and nasal obstructions are the common presenting symptoms, which are identical to those produced by primary tumours in the same area.^1,2,16^ Recurrent profuse epistaxis appears to be specific to haemangiomas and certain metastatic tumours, including renal cell carcinoma and melanoma.^17^ Frequent nasal bleeding from these metastatic tumours are known to be associated with their hypervascularity. In addition to these tumours, metastatic HCC might be a candidate for recurrent profuse epistaxis owing to the high vascularity of the tumour as well as coagulopathy owing to underlying liver cirrhosis.^18^ The present patient, however, had no sinonasal symptoms when he was referred for headache and the nasal cavity mass was incidentally found on brain MRI scan. Nasal obstruction, facial pain and rhinorrhea had developed during follow-up as the size of the mass rapidly increased. Thus, as in the present case, the only clue of metastasis might be a history of a primary tumour elsewhere.

In this case, the metastatic HCC showed high *T*_2_ signal intensity with no restricted diffusion on DWI. Virtually all sinonasal tumours are highly cellular, with relatively little intracellular and intercellular water.^19^ As a result, the majority of these tumours have intermediate signal intensity on *T*_2_ weighted images.^19^ It is rare for sinonasal malignant tumours to have inherently high *T*_2_ weighted signal intensity. Such high *T*_2_ weighted signal intensity may occur with benign or low-grade minor salivary gland tumours, schwannomas, haemangiomas and polypoid tumours such as inverted papillomas.^20^ Technically feasible in the head and neck regions, the addition of DWI increases detection of malignant lesion and is useful for differentiating both solid from cystic lesions and benign from malignant lesions.^21^ Mostly, malignant lesions have lower ADC values compared with benign lesions. In a retrospective study of 33 patients with 17 benign and 16 malignant head and neck lesions, an optimal ADC threshold of 1.3 × 10^−^^3 ^mm^2^ s^−1^ was established for diagnosis of malignant tumours from benign lesions.^22^ In the present patient, the mass showed high signal intensity with intermediate signal foci on *T*_2_ weighted images and restricted diffusion of the tumour was not apparent (average ADC value 1536.01 mm^2^ s^−1^) on DWI. This finding probably related to the differentiation of the metastatic HCC, as it is known that histopathological differentiation of HCC is inversely correlated with the ADC value.^23^ The metastatic HCC presented here was moderately differentiated. This suggested that metastatic HCC may have varying *T*_2_ signal intensity with a wide range of ADC values, and may mimic benign tumours in the nasal cavity. Further investigation should be performed with a larger case series.

Metastasis to the nasal cavity is usually associated with advanced disease and early mortality. As most extrahepatic HCC occurs in patients with an advanced intrahepatic stage of tumour,^3^ metastasis to the sinonasal region is also associated with advanced disease and early mortality.^3,24,25^ The mean survival time of patients is reported to range from 4 weeks to 26 months after the identification of sinonasal metastasis.^18,26,27^ In our review of the literature,^6–8,16,26–32^ most patients were dead less than 2 months after diagnosis of the nasal cavity metastasis. Three of 13 reported patients died of terminal hepatic failure, one of sudden respiratory failure and one of multiple metastases; the remaining four cases were in the following 8 to 15 months. Various treatments for metastatic HCC to the sinonasal cavity have been reported, including surgical resection, palliative radiotherapy and transcatheter arterial embolisation to control nasal bleeding. The treatment should be selected on an individual basis and the purpose of treatment.^8,27,32^

## Conclusions

In conclusion, although metastatic HCC to the nasal cavity is rare and the imaging findings are rather nonspecific, clinicians and radiologists should be aware of this unusual presentation because of its poor prognosis and the possibility of rapid deterioration in the setting of underlying HCC. We present a rare case of metastatic HCC to the nasal cavity with MRI scan including DWI.

## Learning points

1. Metastasis to the nasal cavity of HCC is extremely rare. But when it occurs, it usually associated with advanced disease and early mortality.
2. Most of sinonasal malignant tumours are highly cellular, with relatively little intracellular and intercellular water, so they have intermediate signal intensity on *T*_2_ weighted image and have lower ADC values compared with benign tumours.
3. But, even sinonasal metastatic cancer of HCC has high signal intensity on *T*_2_ weighted images, and restricted diffusion of the tumour was not apparent on DWI depending on histopathological differentiation.

## Consent

Written informed consent for the case to be published (includingimages, case history and data) was obtained from the patient(s) for publication of this case report, including accompanying images.
